# Improved detection of clinically relevant fusion transcripts in cancer by machine learning classification

**DOI:** 10.1186/s12864-023-09889-y

**Published:** 2023-12-18

**Authors:** Völundur Hafstað, Jari Häkkinen, Malin Larsson, Johan Staaf, Johan Vallon-Christersson, Helena Persson

**Affiliations:** 1 Faculty of Medicine, Department of Clinical Sciences Lund, Oncology, Lund University Cancer Centre, Lund, Sweden; 2 Department of Physics, Chemistry and Biology, National Bioinformatics Infrastructure Sweden, Science for Life Laboratory, Linköping University, Linköping, Sweden; 3 Faculty of Medicine, Department of Laboratory Medicine, Translational Cancer Research, Lund University Cancer Centre, Lund, Sweden

**Keywords:** Fusion transcript, Gene fusion, Cancer genomics, Tumor biology, Precision medicine, Machine learning, Microhomology, Kinase

## Abstract

**Background:**

Genomic rearrangements in cancer cells can create fusion genes that encode chimeric proteins or alter the expression of coding and non-coding RNAs. In some cancer types, fusions involving specific kinases are used as targets for therapy. Fusion genes can be detected by whole genome sequencing (WGS) and targeted fusion panels, but RNA sequencing (RNA-Seq) has the advantageous capability of broadly detecting expressed fusion transcripts.

**Results:**

We developed a pipeline for validation of fusion transcripts identified in RNA-Seq data using matched WGS data from The Cancer Genome Atlas (TCGA) and applied it to 910 tumors from 11 different cancer types. This resulted in 4237 validated gene fusions, 3049 of them with at least one identified genomic breakpoint. Utilizing validated fusions as true positive events, we trained a machine learning classifier to predict true and false positive fusion transcripts from RNA-Seq data. The final precision and recall metrics of the classifier were 0.74 and 0.71, respectively, in an independent dataset of 249 breast tumors. Application of this classifier to all samples with RNA-Seq data from these cancer types vastly extended the number of likely true positive fusion transcripts and identified many potentially targetable kinase fusions. Further analysis of the validated gene fusions suggested that many are created by intrachromosomal amplification events with microhomology-mediated non-homologous end-joining.

**Conclusions:**

A classifier trained on validated fusion events increased the accuracy of fusion transcript identification in samples without WGS data. This allowed the analysis to be extended to all samples with RNA-Seq data, facilitating studies of tumor biology and increasing the number of detected kinase fusions. Machine learning could thus be used in identification of clinically relevant fusion events for targeted therapy. The large dataset of validated gene fusions generated here presents a useful resource for development and evaluation of fusion transcript detection algorithms.

**Supplementary Information:**

The online version contains supplementary material available at 10.1186/s12864-023-09889-y.

## Background

Mutational processes in cancer cells create unique genomes with genetic changes that range from single base pairs to larger structural variants such as copy-number changes and translocations of chromosome segments. Structural alterations that lead to juxtaposition of sequences from two different genes can result in a fusion gene. These can encode chimeric proteins or alter the regulation of gene expression through promoter-swapping. We have previously shown that non-coding and out-of-frame fusions can deregulate the expression of intronically encoded small non-coding RNAs including microRNA (miRNA) and small nucleolar RNA (snoRNA) [1–3]. There are many well-established examples of oncogenic gene fusions and some have been successfully exploited as targets for therapy. This includes *BCR-ABL* in chronic myelogenous leukemia (CML), *ALK* and *ROS1* kinase fusions in non-small cell lung cancer, as well as *TRK* fusions in solid cancers [4–7]. The highly specific presence of gene fusions in cancer cells can also be used as somatic tumor fingerprints to trace residual disease or developing therapy resistance in cancer patients by PCR-based methods [8].

Gene fusions can be detected using high-throughput methods including RNA sequencing (RNA-Seq) and whole genome sequencing (WGS) or using commercially available panels for known clinically relevant fusion partners. Identification of fusion transcripts in RNA-Seq data offers advantages over WGS such as functional information about gene and fusion transcript expression, as well as a lower cost per sample. However, fusion detection algorithms often output false positive fusion predictions due to, e.g., misalignment of RNA-Seq data [9, 10]. Furthermore, genomic breakpoints in the introns of fusion partners cannot be identified from spliced fusion transcripts. WGS can provide information about the exact location of genomic breakpoints but does not distinguish between expressed and non-expressed gene fusion events and may therefore identify many non-functional genomic rearrangements. The combination of RNA-Seq and WGS can be used to accurately identify genuine gene fusion events; if the chimeric mRNA sequence of a fusion transcript is supported by discordant read pairs at the genomic level this is a strong indicator that it is a true fusion. Additional support can be added by detection of the exact genomic breakpoints.

Next-generation sequencing is gradually becoming a part of clinical practice in oncology. Efforts include tumor profiling by transcriptome analysis in breast and bladder cancer [11, 12] and combined analysis of DNA and RNA for cancer gene panels to select treatment in metastatic cancer [13]. Although the combination of WGS and RNA-Seq could provide important prognostic and treatment-predictive information, these methods are still costly and the data analysis is time-consuming. New methods for data analysis continue to be developed and there is not always a consensus as to the best way to perform analyses. This may be especially true for fusion transcripts, where a large number of tools have been developed but produce very different results when applied on the same dataset [9, 10, 14]. When RNA-Seq is included in clinical routine for profiling of tumor subtype, prognostication, or other purposes, it produces data that should be put to the best possible use for the patient. Better fusion prediction algorithms with lower rates of false positives and false negatives are needed to accurately and rapidly identify clinically relevant fusions. Better methods for prediction would also improve the analysis of rearrangements in cancer sample cohorts for research purposes. Currently it is unclear what the main sources of false positive fusion transcript predictions are, how much they each contribute, and how this knowledge could best be used for accurate and sensitive identification of fusion genes.

Here, we have analyzed to what extent reads supporting fusion transcripts detected in RNA-Seq data can be found in WGS data and which features characterize these expressed gene fusion events. We have used this knowledge to construct machine learning classifiers to predict true positive fusions from features available from fusion transcript predictions based exclusively on RNA-Seq data. We furthermore show that this can improve fusion transcript predictions and impact the biological interpretation of fusion transcript data. Mechanistic clues from the validated fusions suggest that many gene fusions are created by intrachromosomal amplification events where the genomic breakpoints are characterized by microhomology. Finally, we propose that the combination of fusion transcript prediction from RNA-Seq data with machine learning-based filtering to increase the rate of true positives could facilitate the detection of clinically relevant gene fusion events.

## Results

### Fusion transcript validation in whole genome sequencing data

**Fig. 1:**
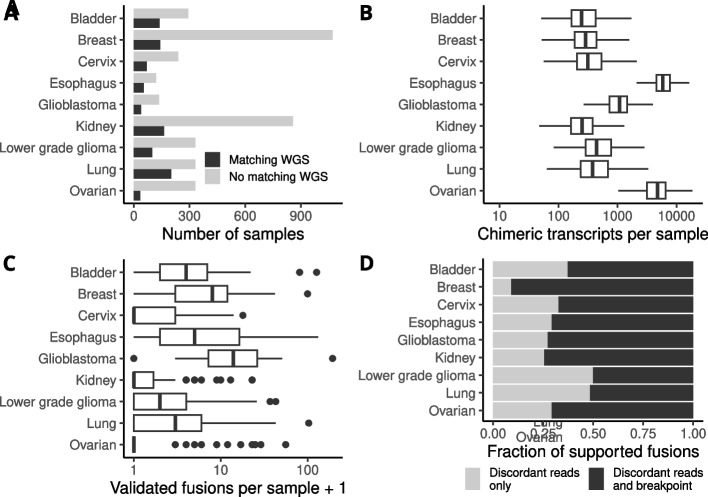
Overview of tumor samples and predicted and validated fusion transcripts. **A** Number of samples that have RNA-Seq data with and without matched WGS data for validation of fusion transcripts. **B** Detected fusion transcripts per sample and cancer type after removing fusions flagged by FusionCatcher as likely false positives. **C** Distribution of the number of validated gene fusions per sample and cancer type. **D** Fraction of the validated fusion genes that had discordant read pairs and at least one determined genomic breakpoint. The three different types of kidney cancer were pooled for plotting

To create a dataset where we could identify true and false positive gene fusion events, we ran the fusion prediction software FusionCatcher for sensitive fusion detection on 4760 RNA-seq samples from 11 diverse cancer types in The Cancer Genome Atlas (TCGA) cohort. Included tissues were BRCA, breast invasive carcinoma; BLCA, bladder urothelial carcinoma; CESC, cervical squamous cell carcinoma and endocervical adenocarcinoma; ESCA, esophageal carcinoma; GBM, glioblastoma multiforme; KICH, kidney chromophobe carcinoma; KIRC, kidney renal clear cell carcinoma; KIRP, kidney renal papillary cell carcinoma; LGG, brain lower grade glioma; LUAD, lung adenocarcinoma, and OV, ovarian serous cystadenocarcinoma. Matched WGS data was available for 910 (19%) of these samples (Fig. 1A). FusionCatcher identified approximately 4.9 million fusion transcripts corresponding to 1.9 million unique sample-partner gene combinations across all RNA-Seq samples. After filtering to remove fusion transcripts flagged as likely false positives (see Methods), approximately 865,000 putative fusions remained in samples with matched WGS data (Fig. 1B).

In order to estimate the rates of true and false positive fusion transcripts, we developed a bioinformatic pipeline to validate gene fusions found in mRNA at the DNA level using matched WGS data [15]. The pipeline consists of a series of scripts that extract, filter and process discordant read pairs in WGS data which support fusion transcripts that were found in RNA-Seq data by FusionCatcher. When discordant read pairs were found, we also attempted to identify a genomic breakpoint by searching for nearby reads with high-quality soft-clipped ends. Soft-clipped ends that passed filtering were locally aligned to the region of the other fusion partner that contained the discordant read. We applied this pipeline to WGS data for validation of the predicted fusion transcripts. We found discordant read pairs supporting 4237 fusion transcripts, and 3049 (72%) of these validated fusion events were further supported by at least one identified breakpoint (Fig. 1C, D, Additional files 1-2). On average, 4.7 fusions were validated per sample with large differences between cancer types. Kidney cancer had the fewest validated fusions per sample (0.8) and glioblastoma had the most (21.6). This observation was not reflected in the number of predicted fusion transcripts per sample, indicating that certain cancer types have higher levels of false positive fusions than others.

To validate the accuracy of our pipeline, we also applied it to putative fusion transcripts detected in normal tissue samples from TCGA. As fusions typically arise from the unstable nature of cancer genomes, we expected to find few or no genuine fusion transcripts in normal tissue. Only 12 of 26,847 putative fusions detected in RNA-Seq data for 85 normal tissue samples had supporting discordant read pairs in matching WGS data. Among these, 3 were detected in a single esophageal sample and 2 were detected in a single breast sample. Overall, fusions detected in normal tissue samples were 20x less likely to be supported by matching WGS data. These results indicate that the validation pipeline is highly specific, with few false positives.

To demonstrate that the discordant read pairs we identified were not stochastic, we also conducted an unbiased search for similar read pairs in WGS data for all genes, regardless of whether or not they were implicated in fusion events. An average of 203 discordant read pairs were detected per tumor sample, but the genes involved in these discordant read pairs had a much lower diversity index compared to fusion transcripts validated with our pipeline (Shannon-Wiener diversity index 0.02 *vs* 2.01). For comparison, the diversity index of the non-validated fusion transcripts was 0.57. The TCGA breast cancer cohort has WGS data for 104 matched normal tissue samples, and we applied the unbiased search to those as well, hypothesizing that discordant read pairs found in normal tissues would likely be false positives. Approximately 45% of all unbiased gene pairs linked by discordant reads in tumor WGS data were also found in the matched normal sample. Similarly, 67% of all unbiased gene pairs detected in normal tissues were also found in the matched tumor sample. Together, these results suggest that discordant read pairs that do not necessarily represent true genomic rearrangements can be found in tumor WGS data. They are dominated by a few reoccurring gene combinations and caution should be exercised when interpreting discordant read pairs as gene fusions based on WGS data only. As a contrast, the average intersection over union for fusion transcripts detected in matched normal and tumor RNA-Seq samples was only 2.7% with a standard deviation of 1.7. RNA-Seq data from matched normal tissue is therefore insufficient as a filter to remove likely false positive fusion transcripts.

### The fraction of validated fusion transcripts does not depend on WGS depth

**Fig. 2:**
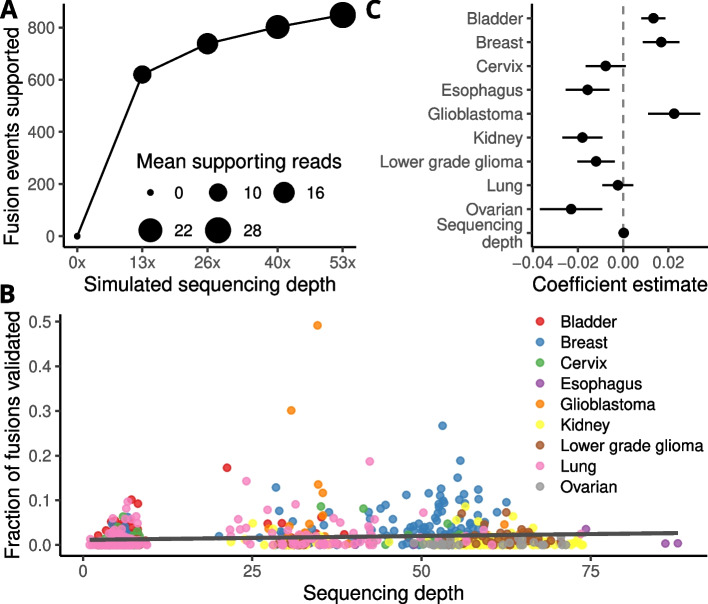
WGS depth has a limited effect on fusion transcript validation rate. **A** Reads from breast tumors with high-coverage WGS were subsampled to 0.25, 0.5 and 0.75 of the original coverage and used for validation. Most validated fusions are still detected at a low coverage of 13x. **B** Fraction of fusion transcripts validated per sample plotted against WGS sequencing depth showing a weak positive correlation.** C** Compared to cancer type, sequencing depth has a negligible effect on the fraction of validated fusion transcripts in linear modeling

As shown above, the fusion transcript validation rate was strikingly low and varied considerably between different cancer types. The sequencing depth of the WGS data also differed between samples and cancer types, from ~7x for bladder cancer to ~60x for kidney cancer. To test how sensitive the WGS validation pipeline is to sequencing depth, we subsampled reads from high-coverage (>40x) breast cancer WGS samples at a rate of 0.25, 0.5 and 0.75 of the original coverage and used those for validation. Approximately 73% of all validated fusion transcripts were still detected at the lowest subsampling rate, simulating a sequencing depth of ~13x (Fig. 2A). We also examined the fusion validation ratio of each sample and compared it to the sequencing depth (Fig. 2B). Although there was a weak positive correlation between fusion validation rates and sequencing depth (*r* = 0.13, *p* = 1.5 x 10^-4^, Pearson’s product moment correlation), this trend vanished when we included the effect of each cancer type in a linear model (Fig. 2C). Similarly, we saw no added effect from tumor purity. Sequencing depth therefore appears to have a relatively small effect on the sensitivity of our fusion validation pipeline.

### Comparison of validated fusion transcripts to established fusion databases

To further evaluate the performance of our validation pipeline, we compared our results to the TumorFusions database [16]. This database lists high-confidence fusion events detected in TCGA and includes WGS validation status where available. The overlap between this database and our FusionCatcher results consisted of 585 fusion events in 210 samples with WGS data (Table 1). Our fusion validation pipeline found WGS evidence supporting 405 of these fusion transcripts, but only 161 of them (40%) were previously reported as validated by WGS. Comparing these two groups of fusion events, we found that genes in fusion events labeled as validated in the TumorFusions database had significantly lower expression than genes in non-validated events (*p* = 0.049 and *p* = 1.4 x 10^-9^ for 3' and 5' partners respectively, Student’s *t*-test). The 3' fusion partners were also significantly shorter in genes in non-validated events (*p* = 2 x 10^-3^, Student’s *t*-test). The TumorFusions database contains 180 fusions that were detected by FusionCatcher in the RNA-Seq data but could not be validated in WGS data by our pipeline. Strikingly, only 22 of these were labeled as being validated by WGS data in the TumorFusions database. Our analysis found an additional 1704 fusion transcripts in these samples that were validated at the DNA level but were not listed in the TumorFusions database. Furthermore, 107 fusion events listed in the TumorFusions database were filtered out in our analysis due to their high probability of being false positives. These results demonstrate that our validation pipeline is highly sensitive, and suggest that it can provide valuable information to complement existing gene fusion databases. The discrepancies between our set of fusion transcripts and the TumorFusions database likely depend on a number of factors including differences in software, filtering criteria, genome assembly, and transcript annotation.

**Table 1:** Overlap between fusion events detected by our validation pipeline and the TumorFusions database

	TumorFusions database	
	Validated	Not validated
Validated by pipeline	161	244
Not validated by pipeline	22	158

### A supervised machine learning model can predict true positive fusion transcripts

**Fig. 3:**
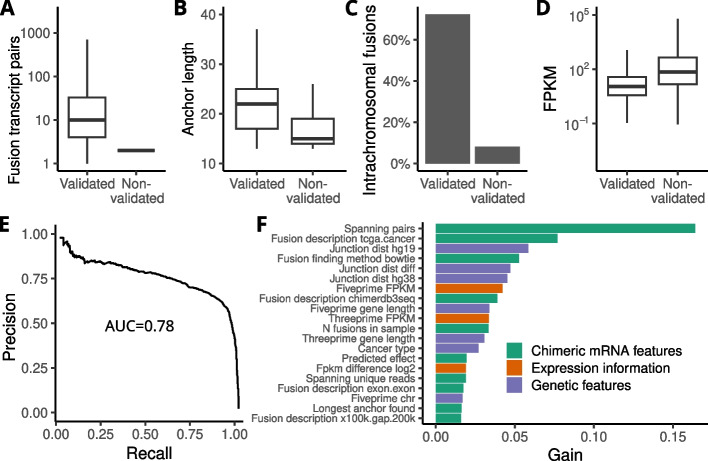
Characteristics of validated fusions and classifier performance for prediction of true positive fusion transcripts. **A** Compared to non-validated fusion transcripts (*n*= 861593), validated fusion transcripts (*n*=4237) had more supporting read pairs in RNA-Seq data, **B** longer anchor length for RNA-Seq reads mapped to the fusion junction, **C** a higher fraction of intrachromosomal fusions, and **D** lower average expression. **E** Precision-recall curve for classifier performance in prediction of true positive fusion transcripts for an independent cohort of 249 breast tumors. **F** Top predictive features color-coded by source of information

To identify possible ways to improve the accuracy of fusion transcript prediction we then compared the characteristics of true and false positive fusion events in TCGA samples with paired RNA-Seq and WGS data. While there was no single feature that could discriminate perfectly between true and false positive fusion transcripts, they did differ in many ways. Unsurprisingly, validated fusions generally had greater support at the transcript level. This was apparent in the average number of supporting read pairs (Fig. 3A) and anchor length (Fig. 3B) being significantly larger for validated fusions (both *p* < 2 x 10^-16^, Student’s *t*-test). Of the transcripts detected by FusionCatcher, approximately 12% were events between genes located on the same chromosome (intrachromosomal events). In contrast, 71% of validated events were found to be intrachromosomal (Fig. 3C, p < 2 x 10^-16^, χ^2^ test). In addition, validated fusions had on average lower expression than non-validated (Fig. 3D). As we have previously reported miRNA and snoRNA host genes to be enriched in fusion events [2, 3], we modeled the probability of a gene being part of a validated fusion event against its status as miRNA host, snoRNA host, and gene length in a logistic regression model. Because of the large differences in expression and gene length between protein-coding and non-coding genes, the analysis was limited to genes annotated by GENCODE as protein-coding. The host status of a gene positively influenced its likelihood of being part of a validated fusion event, with size-adjusted odds ratios of 1.37 for miRNA hosts and 1.67 for snoRNA hosts (*p* = 3.6 x 10^-4^ and *p* = 1.5 x 10^-8^, respectively).

Based on these observed differences, we hypothesized that it may be possible to use machine learning to train a classifier for prediction of true and false positive fusions. To be a useful addition to conventional software for identification of fusion transcripts, such a classifier should only use information that is available also for samples without WGS data. After evaluating different machine learning algorithms, we constructed a light gradient-boosting machine (LightGBM) classifier. The classifier was trained on fusion transcripts in samples with matched WGS and RNA-Seq data using only features available on the RNA level or from common sources of genomic annotation, so that the predictor could later be applied to samples without WGS data. The WGS validation status of each fusion event was used as the ground truth for the fusion status. The features used to train the model can be broadly divided into three categories:

1. Chimeric mRNA features, e.g. number of spanning reads detected and longest anchor found.
2. Expression information for the mRNA, fragments per kilobase of exon model and million reads (FPKM), and relative expression between the two fusion partners.
3. General genetic features including spatial information, gene status in the COSMIC database, overlapping repetitive regions, and miRNA host status.

The full list of features used is available in Additional file 3. The model was only trained on fusions that passed initial filtering criteria, i.e. did not include banned tags or common mapping reads between the two partner genes. Since the data was highly imbalanced with only around ~1% true positives, we trained the model for an optimal combination of area under the precision-recall curve and f1 score when tuning hyperparameters. Model performance in training was evaluated using a leave-one-group-out cross-validation, with each cancer type in a separate group. This was done to ensure robust performance across multiple cancer types where the fusion validation rates differed greatly. The winning model’s precision and recall metric estimates were similar, reaching 0.86 and 0.85, respectively, at a classification threshold of 0.2. To measure of the robustness of the classifier, the final model was evaluated on an independent tumor cohort. We used a set of 249 triple-negative breast cancer (TNBC) samples from the SCAN-B study with matched RNA-Seq and WGS data [17]. The model achieved an area under the precision-recall curve of 0.78 on the test data (Fig. 3E) and an f1 score of 0.73 with precision 0.74 and recall 0.71. A Cohen’s Kappa value of 0.71 for the test data indicates strong agreement between the classifier and the results of the WGS validation pipeline. The confusion matrix is shown in Table 2. Extracting feature importance revealed that the number of spanning read pairs supporting a fusion transcript contributed the most to the classification, but that features from all three categories contributed to the prediction (Fig. 3F).

**Table 2:** Confusion matrix for the breast cancer test dataset

		**Prediction**	
		True	False
**Truth**	True	864	350
	False	308	26072

### A machine learning-based filtering approach outperforms classical filtering methods

**Fig. 4:**
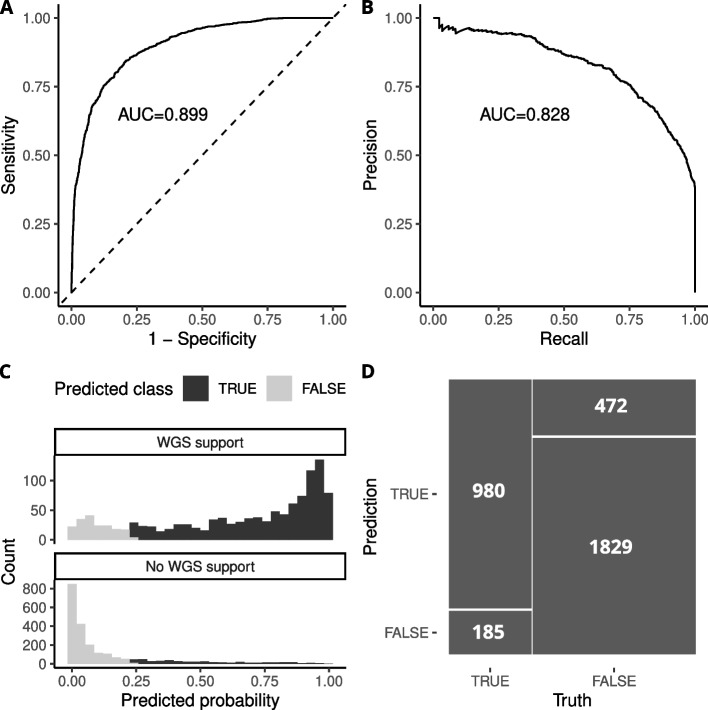
Performance of a machine learning classifier for fusions detected by Arriba in the BRCA and LUAD TGA cohorts. **A** The classifier achieved an area under the ROC curve of 0.90 and **B** area under the precision-recall curve of 0.83. **C** Predicted classification probability of each fusion event vs actual WGS validation status, at a classification threshold of 0.23. **D** Confusion matrix of the classifier when applied to testing data at a classification threshold of 0.23

To assess the benefits of using a machine learning-based approach to filter fusion transcripts, we trained a second classifier on fusions detected by Arriba in the BRCA and LUAD TCGA cohorts (see Methods). The classifier achieved an area under the ROC curve of 0.90 and an area under the precision-recall curve of 0.83 in the test dataset, and precision and recall metrics of 0.68 and 0.84 respectively at an optimal classification threshold of 0.23 as determined by Youden’s J statistic (Fig. 4A-D). We applied this classifier to fusions detected by Arriba in the same set of 249 TNBC SCAN-B samples that were used for evaluation of the previous model. In this dataset the model achieved an area under the precision-recall curve of 0.79 and an area under the ROC curve of 0.76 (Table 3). This demonstrates that the models can be applied to cohorts that are independent of the training data and still have robust performance, and that this is also true for fusion transcripts from two different fusion callers.

**Table 3:** Performance metrics for machine learning-based filtering compared to classical filtering methods on fusions detected with Arriba in the BRCA and LUAD TCGA cohorts

**Metric**	**Classifier** **(testing data)**	**High confidence**	**High+med confidence**	**RNA disc.** **mates > 2**	**High + med confidence and** **RNA disc. mates > 2**	**In-frame only**
precision	0.68	0.43	0.44	0.37	0.47	0.53
recall	0.84	0.46	0.71	0.83	0.63	0.16
specificity	0.80	0.69	0.54	0.27	0.64	0.93
accuracy	0.81	0.61	0.60	0.46	0.64	0.67
f1 score	0.75	0.45	0.54	0.51	0.54	0.24
kappa	0.60	0.15	0.22	0.08	0.25	0.11
log loss	0.39	-	-	-	-	-
roc auc	0.90	-	-	-	-	-
pr auc	0.83	-	-	-	-	-
brier score	0.12	-	-	-	-	-

To determine the usefulness of our machine learning-based filtering approach, we compared our Arriba-based model to the performance metrics of several different “classical” filtering approaches:

1. Keeping fusion events labeled as “high” confidence by Arriba
2. Keeping “high” and “medium” confidence fusions
3. Keeping fusion transcripts supported by 3 or more discordant mates
4. Keeping fusion events that pass 2. and 3.
5. Keeping only in-frame fusions

These filtering methods were applied to the combined training and test data of the model (Table 3) and to the TNBC SCAN-B data (Table 4), and performance metrics derived in the same manner as the ML classifier. Probability-based metrics such as ROC AUC were excluded as standard filtering produces no probability value. Our machine learning classifier outperformed all approaches in nearly every metric in both TCGA and SCAN-B cohorts. The classical filtering approach that performed the best was to keep fusions tagged as high or medium confidence by Arriba which achieved an f1 score of 0.54 in the TCGA data and 0.70 in the SCAN-B data. The corresponding f1 scores for our classifier were 0.75 and 0.72, respectively. For all other performance metrics, our model performed on-par with or better than the best classical filtering approach. The FusionCatcher-based model similarly outperformed classical filtering methods (Additional file 4). These results show the benefits of choosing a data-driven approach to fusion transcript filtering.

**Table 4:** Performance metrics for machine learning-based filtering compared to classical filtering methods on fusions detected with Arriba in an independent fusion dataset of 249 TNBC samples from the SCAN-B cohort

**Metric**	**Classifier**	**High confidence**	**High+med confidence**	**RNA disc. mates > 2**	**High + med confidence and RNA disc. mates > 2**	**In-frame only**
precision	0.74	0.70	0.68	0.63	0.72	0.69
recall	0.70	0.54	0.72	0.69	0.54	0.20
specificity	0.67	0.68	0.55	0.46	0.72	0.88
accuracy	0.69	0.60	0.64	0.59	0.62	0.49
f1 score	0.72	0.61	0.70	0.66	0.62	0.31
kappa	0.37	0.22	0.27	0.15	0.25	0.07
log loss	0.83	-	-	-	-	-
roc auc	0.76	-	-	-	-	-
pr auc	0.79	-	-	-	-	-
brier score	0.26	-	-	-	-	-

### Predicted true positive fusion transcripts reflect the biology of validated fusions

Having demonstrated that machine learning classifiers can be used to improve fusion transcript prediction with good precision and recall as judged by WGS data validation, we applied our FusionCatcher-based model to fusions detected in TCGA tumors with only RNA-Seq data. This expanded the set of tumor samples available for analysis from 910 to 4760. After pre-filtering as before, we applied our classifier to 3.5 million fusion transcripts detected in these samples. The classifier tended to be conservative, with lower ratios of fusions predicted to be true compared to the WGS validation. To evaluate if the resulting 13,376 predicted true fusion transcripts resembled the smaller set of validated fusions in terms of genes and pathways, we performed a gene set over-representation analysis on the genes involved in these events. To take recurring fusion genes into account, we based the over-representation analysis on the binomial distribution (see Methods). The 5' and 3' fusion partners were analyzed separately within each cancer type. A principal component analysis (PCA) of the enriched gene sets revealed that validated fusion events and those predicted by the machine learning classifier in the same samples generally clustered close together in a high-dimensional space. We then expanded the analysis to include predicted true positive fusion transcripts from all samples, including fusion events in samples that only had RNA-Seq data. These results were compared to enrichment results for the total, unselected FusionCatcher output. The enrichment results for both groups of predicted true fusion transcripts clustered closer to validated fusions and further away from the total FusionCatcher output (Additional file 5). This indicates that enrichment analysis of fusions that our classifier predicts to be true positive events gives results that are biologically more similar to validated fusions – a potentially critical feature when looking at the functions of fusions.

### Identified breakpoints in validated fusions provide mechanistic information

**Fig. 5:**
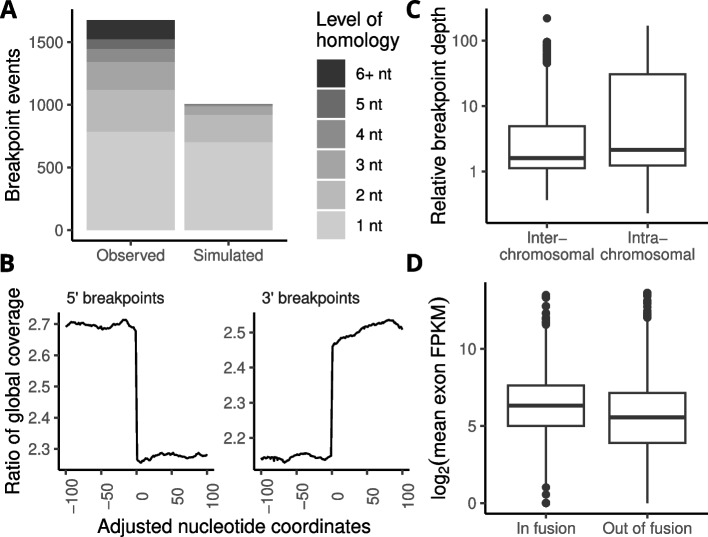
Genomic breakpoints provide information about mechanisms creating gene fusions. **A** Microhomology between fusion partners was significantly more common at genomic breakpoints than in control regions. **B** Nucleotide positions flanking genomic breakpoints had higher sequencing depth in WGS data relative to the genome average with a distinct drop on the side of the breakpoint that was not in the fusion transcript. **C** The higher relative sequencing depth was especially pronounced in intrachromosomal fusions, indicating amplification as a possible mechanism.** D** Exons included in validated fusion transcripts (n=1295) had higher expression than excluded exons of the same gene and sample in the SCAN-B cohort where exon-level expression was available

The pipeline we developed for validation of fusion transcripts also identified a genomic breakpoint in a majority of the cases. This type of information is important for guiding experimental validation of fusion events, and can also be used to explore the mechanisms that create gene fusions. The majority of breakpoints (81%) were found within introns, but this percentage was lower than what would be expected based on intron length alone. Notably, breakpoints located within coding sequences were more common than expected when considering their length, while breakpoints inside untranslated regions (UTRs) were less common than expected (*p* < 2 x 10^-16^, χ^2^ test). Approximately 70% of breakpoints overlapped repetitive elements, the most common of those being L1 and L2 long interspersed nuclear elements (LINEs). Previous studies have suggested that a significant portion of fusions might arise due to alternative non-canonical end-joining (Alt-NHEJ) [18]. We analyzed the genomic sequences immediately up- and downstream of genomic breakpoints for validated fusion events with at least one identified breakpoint. As a control we simulated additional breakpoints in the same genomic regions as observed breakpoints and compared the levels of microhomology found there (Fig. 5A). We observed significantly more microhomologous sequences at the observed breakpoints compared to simulated breakpoints in every cancer type (*p* < 2 x 10^-16^, Mann-Whitney U test). This is a pattern that would be compatible with Alt-NHEJ as a DNA repair mechanism for creation of gene fusions [19]. Gene fusions can be a result of chromosomal translocations or, alternatively, amplification or deletion of chromosome segments. Intrachromosomal fusions were common among the validated fusion transcripts, and these could be created by amplification or deletion. To explore possible mechanisms, we calculated the mean sequencing depth for each nucleotide in the vicinity of fusion breakpoints as a ratio to the global sequencing depth. Nucleotides flanking the breakpoint had on average higher sequencing depth than rest of the genome, with a distinct drop in depth on the side of the breakpoint that was not part of the fusion transcript (Fig. 5B, Additional file 6). This was especially pronounced in intrachromosomal fusions, indicating that these fusions arise from genomic amplification events (Fig. 5C). The TNBC cohort from SCAN-B that we used to validate the fusion transcript classifier had available exon-level expression data that we used to compare expression up- and downstream of breakpoints. We observed that the exons involved in fusion transcript had considerably higher expression than the other exons in the same gene. Interestingly, this effect was only observed for the 3' fusion partners, which had on average a 5-fold difference in expression of exon in- and out of fusions (*p* < 2 x 10^-16^, paired Student's *t*-test, Fig. 5D). Although we did observe a statistically significant difference in the 5' fusion partners as well (*p* = 1.95 x 10^-5^, paired Student's *t*-test), the mean difference was considerably smaller (1.16-fold). This effect was strand-independent. The difference in exon expression of 3' fusion genes may indicate altered transcriptional regulation by the promoter of the 5' partner gene.

### Kinase fusions are frequently detected among predicted true positive fusions

**Fig. 6:**
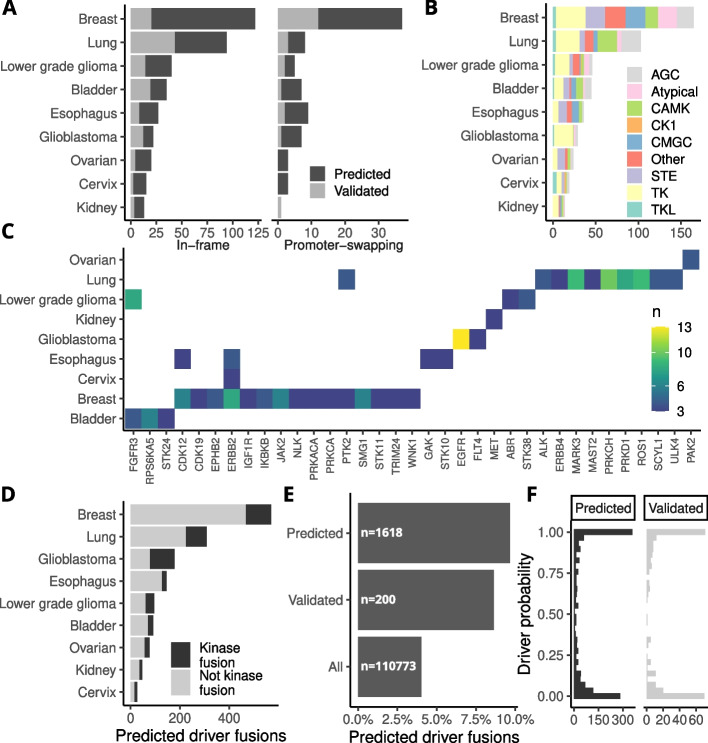
Occurrence of in-frame and promoter-swapping kinase fusions in different cancer types. **A** Number of validated and predicted true positive fusion transcripts involving kinases per cancer type. **B** Number of fusions per kinase family and cancer type for validated and predicted fusion events (see Methods for kinase family abbreviations). **C** In-frame and promoter-swapping kinases involved in 3 or more validated fusion events per cancer type. **D** Number of driver fusions predicted by Oncofuse in validated and predicted true positive fusion events. **E** Percent of fusion events with a high probability (>95%) of being drivers. **F** Driver probability distribution of predicted and validated kinase fusions

Kinase fusions are of particular interest in clinical oncology due to their potential to promote oncogenic activation and cancer progression. Multiple recurrent kinase fusions have previously been identified and are candidates for targeted therapy. We analyzed validated fusion events involving kinases and focused on potentially protein-coding fusions that were either in-frame or had a promoter-swapping event with the kinase as 3' partner. Lung cancer had the highest number of validated in-frame kinase fusions, whereas breast cancer had the most putative promoter-swapping events (Fig. 6A). Fusions involving tyrosine kinases (TK) were overall the most abundant and had a notably higher ratio in glioblastoma samples (Fig. 6B). Interestingly, our analysis revealed that each cancer type had a unique set of kinases in recurrent fusions, with little overlap between cancer types (Fig. 6C). This suggests that the molecular mechanisms that promote the development of kinase fusions are specific to each cancer type, something to take into consideration in the development of targeted therapies. Our analysis pipeline found kinase fusions that could be validated in WGS data which had not previously been reported in these samples, and when our classifier was applied to samples with only RNA-Seq data it greatly expanded the number of potentially actionable kinase fusions. We also analyzed the validated and predicted true positive fusion events with Oncofuse, a software designed to help identify candidate driver fusions [20] (Fig. 6D). The validated and predicted true positive fusion events have a higher fraction of predicted driver fusions than the total set of fusion transcripts (Fig. 6E) and similar distributions of driver probability scores for fusions involving kinases (Fig. 6F).

## Discussion

Gene fusions can create proteins with new properties or alter the expression of both coding and non-coding genes. In clinical management of cancer, identified gene fusions can be used for targeted therapy or to monitor the presence of residual tumor cells or progression. A large number of bioinformatic tools have been developed for identification of fusion transcripts in tumor RNA-Seq data, but the results overlap between these methods is often small and the fractions of false positives and false negatives can be high [9, 10]. This limits the usefulness of fusion transcript predictions for both research and clinical use. Here we have combined independent support from RNA-Seq and WGS data for 910 tumors from TCGA to create a very large set of validated fusion events from 11 different cancer types. We have used these validated fusions to show that 1) true and false positive fusion events have different properties, 2) machine learning classifiers can outperform standard filtering strategies and predict true positive fusion transcripts with high precision and recall, 3) predicted true positive fusion transcripts contain many previously unreported kinase fusions, and 4) identified genomic breakpoints can provide mechanistic information about the creation of fusion genes.

For comparison of true and false positive fusions we focused on features that did not require WGS data, and which could therefore be applied to any set of fusion transcripts. This included expression level, genomic annotation, and information from the fusion prediction software such as supporting reads and inclusion in fusion databases. It is important to note that we removed all fusion transcripts that had been flagged as likely false positives by FusionCatcher already before performing this comparison. This includes e.g. fusions previously found in healthy samples and fusions between adjacent or paralogous genes [21]. Many features differed between true and false positive fusions. For example, true positive fusions had a higher number of supporting reads, lower partner gene expression, and a larger fraction of intrachromosomal fusions. However, none of these differences by themselves were sufficient to allow hard filtering of false positive fusion transcripts.

We therefore developed machine learning classifiers that could integrate many different sources of annotation. With a LightGBM classifier trained on fusion transcripts detected by FusionCatcher we achieved precision and recall metrics of 0.74 and 0.71, respectively, when it was tested on an independent cohort of 249 TNBC samples [17]. These tumors are molecularly heterogenous but are characterized by genomic instability with frequent chromosomal rearrangements [22]. Since false positives outnumber true positives in both datasets this implies that it can drastically reduce the number of predicted fusion transcripts while retaining a majority of the real fusion events. When the classifier was applied to the larger set of TCGA samples that only had RNA-Seq data, the gene sets enriched among predicted true positive fusions were similar to validated fusions while both differed from the total set of fusion transcripts. This indicates that machine learning can be used as a complement to improve fusion transcript prediction, also in datasets that only have RNA-Seq data. Excluding likely false positive fusion predictions would facilitate experimental validation of fusion events and improve the quality of any biological interpretation.

The TCGA RNA-Seq and WGS data that were used here are available upon application, but hosting these data requires substantial storage space (approximately 200 TB just for WGS data for 910 samples from 11 cancer types) and the time needed for data download is a limiting factor. With the resources available to us we had to resort to downloading and analyzing the data in batches and deleting data in between. In the end, we also had to cap data download before we could analyze all available cancer types to be able to finalize a manuscript. For this reason, some interesting cancer types with known driver fusions, such as prostate cancer, were not included in this work. Fusion transcripts from the two newer algorithms Arriba [23] and STAR-Fusion [14] have later become available for TCGA samples through the Genomic Data Commons (GDC) Data Portal [24]. We were therefore able to compare the performance of FusionCatcher with these tools for a limited number of samples. As shown in Additional file 7 the three tools differ in performance with Arriba clearly being the most sensitive software. Fusion prediction algorithm development continues, however, and there are considerable differences between the version of Arriba that was used to generate these publicly available predictions (v1.1.0) and the latest version (v2.4.0). For example, information about fusion transcript reading frame is now available in the output. To evaluate the usefulness of adding a machine learning-based classifier also for another fusion prediction algorithm we therefore ran Arriba v2.4.0 and trained a classifier on BRCA and LUAD, two of the largest TCGA cohorts. When applied to the TNBC cohort, the precision and recall were 0.68 and 0.84, respectively, indicating that machine learning could be broadly applied to improve the quality of fusion transcript predictions. Our comparison with different filtering strategies also showed that a data-driven approach can perform better than classical filters such as number of supporting read pairs or Arriba confidence score.

Since the number of datasets with validated fusion genes is still relatively small, especially for fusions with identified genomic breakpoints, simulated fusion data have been used as an alternative for evaluation of software [9, 10, 14]. Simulated data does not capture all aspects of actual RNA-Seq data, such as artifacts from cDNA synthesis and PCR amplification, read-through transcription, transcribed pseudogenes, or intronic and intergenic reads. With limited real-world data it is also not clear how well simulated fusions reflect the properties of real fusion transcripts. A complementary approach that has been used in other studies is ensemble prediction in RNA-Seq data [10, 14]. The assumption is that true fusions will be detected by more methods and that requiring repeated prediction by several methods will enrich for likely true positive fusion transcripts. Sometimes the matching criteria are quite flexible and include fusions with paralogous genes. This is problematic since such an approach could potentially also enrich for false positive fusion predictions. Our comparison of fusion transcripts from FusionCatcher, Arriba, and STAR-Fusion shows that the ensemble approach can be overly conservative, excluding many true fusions, while not efficiently removing false positives (Additional file 7). The precision for ensemble predictions based on the intersection of Arriba and STAR-Fusion is 62% for TCGA data (BRCA and LUAD combined) and 66% for the TNBC validation set.

Here we provide information on validated fusions as supplementary material, with the intention that they can be used as a valuable complement to simulated fusion transcripts for development and evaluation of fusion-finding algorithms (Additional file 2).

Clinical applications for fusion genes mainly focus on identification of known fusions that involve kinases that can be targeted by available drugs, and screening is practice for some cancer types [25]. While targeted approaches may miss functional fusion events, WGS data is still comparatively expensive to generate and difficult to analyze. RNA-Seq is more amenable to large-scale clinical application and provides gene expression information, which also confirms that identified gene fusions are expressed. Our results show that prediction of fusion transcripts can be combined with a machine learning classifier to accurately identify potentially actionable kinase fusions in tumors with only RNA-Seq data.

## Conclusions

We have demonstrated that prediction of fusion transcripts in RNA-Seq data can be combined with machine learning-based filtering to dramatically increase the rate of true positive fusion events. After training on a limited set of samples with available WGS data, the resulting classifier can be used to improve the accuracy of fusion transcript analyses for both research and clinical purposes. Based on our results, we suggest that this can facilitate studies of tumor biology and the identification of kinase fusions for targeted therapy. The large number of validated gene fusions presented here can also be used as a resource for development and evaluation of fusion transcript prediction software.

## Methods

### Fusion transcript prediction

We used FusionCatcher version 1.00 to analyze all available RNA-Seq data for 11 cancer types in the TCGA database. BAM files with coordinates in GRCh38 were downloaded using the GDC Data Transfer Tool Client. We used custom parameters for FusionCatcher to detect as many putative fusion events as possible. We therefore changed the Length_anchor and Length_anchor2 parameters to "13,14,14,14,14" and "22", respectively, spanning_pairs and spanning_reads to "2,2,2,2,2" and "1,1,1,1,1", respectively, and mismatches_psl to 4. Fusion transcripts flagged by FusionCatcher as likely false positives with the following tags were removed: 1000genomes, 1K<gap<10K, adjacent, ambiguous, duplicates, ensembl_partially_overlapping, gap<1K, gencode_fully_overlapping, gencode_partially_overlapping, gencode_same_strand_overlapping, healthy, m0, multi, non_cancer_tissues, non_tumor_cells, refseq_partially_overlapping, tcga-normal, ucsc_partially_overlapping, banned, bodymap2, cacg, conjoing, cta_gene, ctb_gene, ctc_gene, ctd_gene, distance1000bp, ensembl_fully_overlapping, ensembl_same_strand_overlapping, gtex, hpa, mt, pair_pseudo_genes, paralogs, readthrough, refseq_fully_overlapping, refseq_same_strand_overlapping, rp_gene, rp11_gene, rrna, similar_reads, similar_symbols, ucsc_fully_overlapping, ucsc_same_strand_overlapping. Additional fusion transcripts prediction for SCAN-B TNBC data was performed using Arriba (v2.3) and STAR-Fusion (v1.11). Arriba and STAR-Fusion calls were retrieved for TCGA LUAD and BRCA samples using the GDC Data Transfer Tool Client.

### Fusion transcript validation in WGS data

WGS BAM files for the TCGA project were downloaded from the GDC Legacy Archive using the GDC Data Transfer Tool Client. Fusion junction coordinates were converted to hg19 using LiftOver to match the TCGA WGS data. To validate fusion transcripts at the DNA level, we used our previously described pipeline [15]. In brief, search regions were defined for every fusion event based on the observed fusion junctions for each fusion partner together with the start and end coordinates of the genes. Discordant read pairs mapping to each of the two fusion partners were extracted from the defined regions and subjected to filtering to remove low-quality reads. Genomic breakpoints were subsequently located by extracting high-quality soft-clipped read ends in proximity to detected discordant read pairs, where the soft-clipped end aligns close to the mate read in the discordant pair.

### Global sequencing depth and subsampling

Global sequencing depth of WGS samples was calculated using samtools by fetching the depth of every 100^th^ base in the genome and calculating mean sequencing depth for each sample. Reads were subsampled from breast tumor samples that had depth >40x, at fractions of 0.25, 0.50 and 0.75 of original reads. Subsampling was performed before any other filtering steps using the samtools filtering options *--subsample 0.[fraction] --subsample-seed 123*. Local sequencing depth was calculated for every nucleotide in a region of -100 to +100 bp flanking detected genomic breakpoints using samtools. Genomic coordinates were adjusted to reflect their location relative to the genomic breakpoint and the strand of the fusion gene.

### Feature selection and machine learning

We constructed a supervised LightGBM model for fusion transcripts using their validation status in WGS data as proxy for the truth. Only features that were accessible in RNA-Seq data, such as fusion partner FPKM and spatial information, were selected. The full list of fusion features used for the classifier is available in Additional file 3. As the LightGBM framework can inherently handle missing and categorical data, relatively little preprocessing is required of the input data. The only preprocessing steps taken for our feature set were to encode multi-categorical list columns and to remove zero-variance features. Training was performed on the results of the WGS validation pipeline for each cancer type using a leave-one-group-out cross validation. The three kidney cancer groups (KICH, KIRP and KIRC) were merged into a single kidney group to increase group size. Hyperparameter configuration for all models can be found on GitHub. Hyperparameter combinations selected for tuning were generated via grid search using maximum entropy parameter grid. Hyperparameter tuning of the model was optimized for area under the precision-recall curve and the model with the best performance was selected. Each model was trained for 100 iterations with the early stopping rounds parameter set to 10. Final model performance was evaluated with the area under the precision-recall curve. The model was constructed in R using the tidymodels 1.0.0, bonsai 0.2.1, finetune 1.0.0 and lightgbm 3.3.3 packages. The list of parameters that were tuned while training the fusion predictor, and their values in the final model.

### Construction of an Arriba-based machine learning classifier

We constructed an additional LightGBM classifier for 13860 fusion events detected by Arriba v2.4 in 312 samples in the BRCA and LUAD TCGA cohorts using the results of the WGS validation pipeline as the outcome. During data preprocessing we attempted to use as many of the same features that were used in the FusionCatcher LightGBM classifier. Information specific to the Arriba output data was used as features instead of the FusionCatcher-specific information used previously. A full list of features is included in Additional file 3. Model training was performed on 75% of the data (10394 fusion events). Model performance was assessed during training using a 10-fold cross-validation resampling on the training data. A total of 250 models were trained. The final model was chosen for optimal PR AUC and Brier Score during resampling. Final model performance was assessed by fitting the model to the remaining untouched 25% (3466) fusion events. Hyperparameter configuration for all models can be found on GitHub.

### Exon expression

Exon level expression information was obtained for 249 breast cancer samples in the SCAN-B cohort. For each gene involved in a validated gene fusion, the exons that were part of the fusion transcripts were tagged and their expression compared to the other exons in the gene using a paired Student’s *t*-test.

### Microhomology analysis

To investigate the potential mechanisms underlying gene fusions, we analyzed the presence of microhomology flanking the breakpoint. The genomic sequence directly upstream of the 5' partner breakpoint was compared to the sequence downstream of the 3' partner breakpoint. Breakpoints were labelled as microhomologous if the sequences matched in the first 1 to 5 nucleotides, and as homologous if there were further matches. We also simulated breakpoints randomly in the vicinity (within 1 kb) of both detected breakpoints and calculated homology as before.

### Enrichment analysis

Enrichment analysis was performed for each cancer type and fusion partner separately. The analysis was performed by calculating the proportion of genes in a list that were associated with a particular pathway, to the proportion of all genes in the “universe” that had that annotation. Here, the universe was defined as all expressed genes in the respective cohorts, i.e., genes that had a 95^th^ percentile FPKM expression of 1 or greater. Because the same gene can be involved in multiple fusion events, we based our enrichment test on the binomial distribution (sampling with replacement), as opposed to the traditional hypergeometric distribution (sampling without replacement). The method described in [26] was implemented in R using the pbinom() function and adjustment for multiple testing was done using Benjamini-Hochberg (FDR) correction.

### Kinase fusion analysis

The validated and predicted true positive sets of gene fusions were analyzed to identify in-frame or promoter-swapping fusions involving kinases. The list of human kinases was downloaded from KinHub [27]. Abbreviations for kinase groups: TK, tyrosine kinases; TKL, tyrosine kinase-like; CAMK, calcium/calmodulin-dependent kinases; AGC, kinase group AGC; RGC, receptor guanylate cyclases; STE, sterile/ste20-related; CMGC, CDK/MAPK/GSK3/CDK-Like; CK1, casein kinase 1. The tumor driver potential of these fusions was analyzed using Oncofuse [20].

### Supplementary Information

**Additional file 1.** Number of samples, predicted fusion transcripts and validated fusion transcripts.

**Additional file 2. **Validated fusion genes in TCGA samples.

**Additional file 3.** Fusion transcript features used in machine learning for the FusionCatcher model.

**Additional file 4.** Performance metrics for machine learning-based filtering compared to classical filtering methods on fusions detected with FusionCatcher in an independent fusion dataset of 249 TNBC samples from the SCAN-B cohort.

**Additional file 5.** PCA of gene set over-representation analysis for all fusion genes detected by FusionCatcher, fusion genes validated by our pipeline, and fusion genes predicted by the machine learning classifier in samples with WGS data (predicted) and in all RNA-seq samples (all predicted).

**Additional file 6.** Sequencing depth around genomic breakpoints in WGS data, expressed as a ratio to the genome average.

**Additional file 7.** Comparison of FusionCatcher with Arriba and STAR-Fusion. **A **Sensitivity for individual fusion prediction algorithms and ensemble predictions based on TCGA data (BRCA and LUAD combined). Upset plots for validated fusions in **B **the TCGA data and **C **the TNBC validation set.

## Data Availability

The datasets analyzed during the current study are available from the TCGA Research Network, https://www.cancer.gov/tcga. The sequence data for the SCAN-B datasets analyzed during the current study are not publicly available due to Swedish law, but are available from the corresponding author on reasonable request. The code is available from the following GitHub repositories: https://github.com/VolundurH/Fusion_transcripts and https://github.com/VolundurH/wgs_fusion_pipeline.

## Abbreviations

Alt-NHEJ: Alternative non-homologous end-joining
BLCA: Bladder urothelial carcinoma
bp: base pairs
BRCA: Breast invasive carcinoma
CESC: Cervical squamous cell carcinoma and endocervical adenocarcinoma
CML: Chronic myelogenous leukemia
ESCA: Esophageal carcinoma
FPKM: Fragments per kilobase of exon model and million reads
GBM: Glioblastoma multiforme
kb: kilobases
KICH: Kidney chromophobe carcinoma
KIRC: Kidney renal clear cell carcinoma
KIRP: Kidney renal papillary cell carcinoma
LGG: Brain lower grade glioma
LightGBM: Light gradient-boosting machine
LINE: Long interspersed nuclear elements
LUAD: Lung adenocarcinoma
miRNA: MicroRNA
OV: Ovarian serous cystadenocarcinoma
PCA: Principal component analysis
RNA-Seq: RNA sequencing
SCAN-B: Sweden Cancerome Analysis Network – Breast
snoRNA: Small nucleolar RNA
TCGA: The Cancer Genome Atlas
TNBC: Triple-negative breast cancer
UTR: Untranslated region
WGS: Whole genome sequencing
