# Supplementary material for: Improved detection of clinically relevant fusion transcripts in cancer by machine learning classification
Source: BMC Genomics. 2023 Dec 18;24:783. doi: 10.1186/s12864-023-09889-y (PMC10726539; doi:10.1186/s12864-023-09889-y)
Supplement: Supplementary file 5 — Additional file 5. PCA of gene set over-representation analysis for all fusion genes detected by FusionCatcher, fusion genes validated by our pipeline, and fusion genes predicted by the machine learning classifier in samples with WGS data (predicted) and in all RNA-seq samples (all predicted). [file 12864_2023_9889_MOESM5_ESM.pdf]

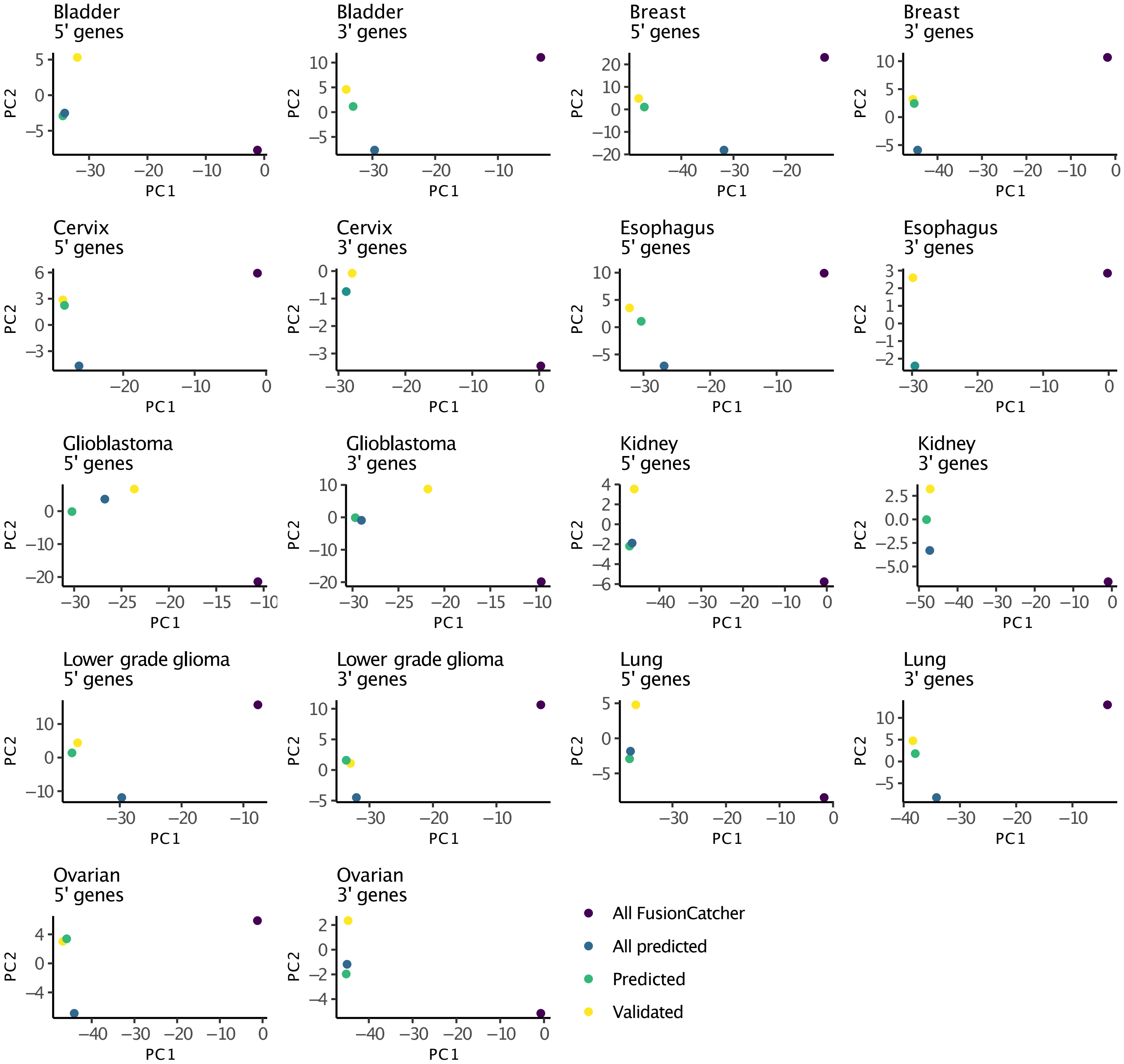

Additional file 5. PCA of gene set over-representation analysis for all fusion genes detected by FusionCatcher, fusion genes validated by our pipeline, and fusion genes predicted by the machine learning classifier in samples with WGS data (predicted) and in all RNA-seq samples (all predicted).
