# Supplementary material for: Improved detection of clinically relevant fusion transcripts in cancer by machine learning classification
Source: BMC Genomics. 2023 Dec 18;24:783. doi: 10.1186/s12864-023-09889-y (PMC10726539; doi:10.1186/s12864-023-09889-y)
Supplement: Supplementary file 6 — Additional file 6. Sequencing depth around genomic breakpoints in WGS data, expressed as a ratio to the genome average. [file 12864_2023_9889_MOESM6_ESM.pdf]

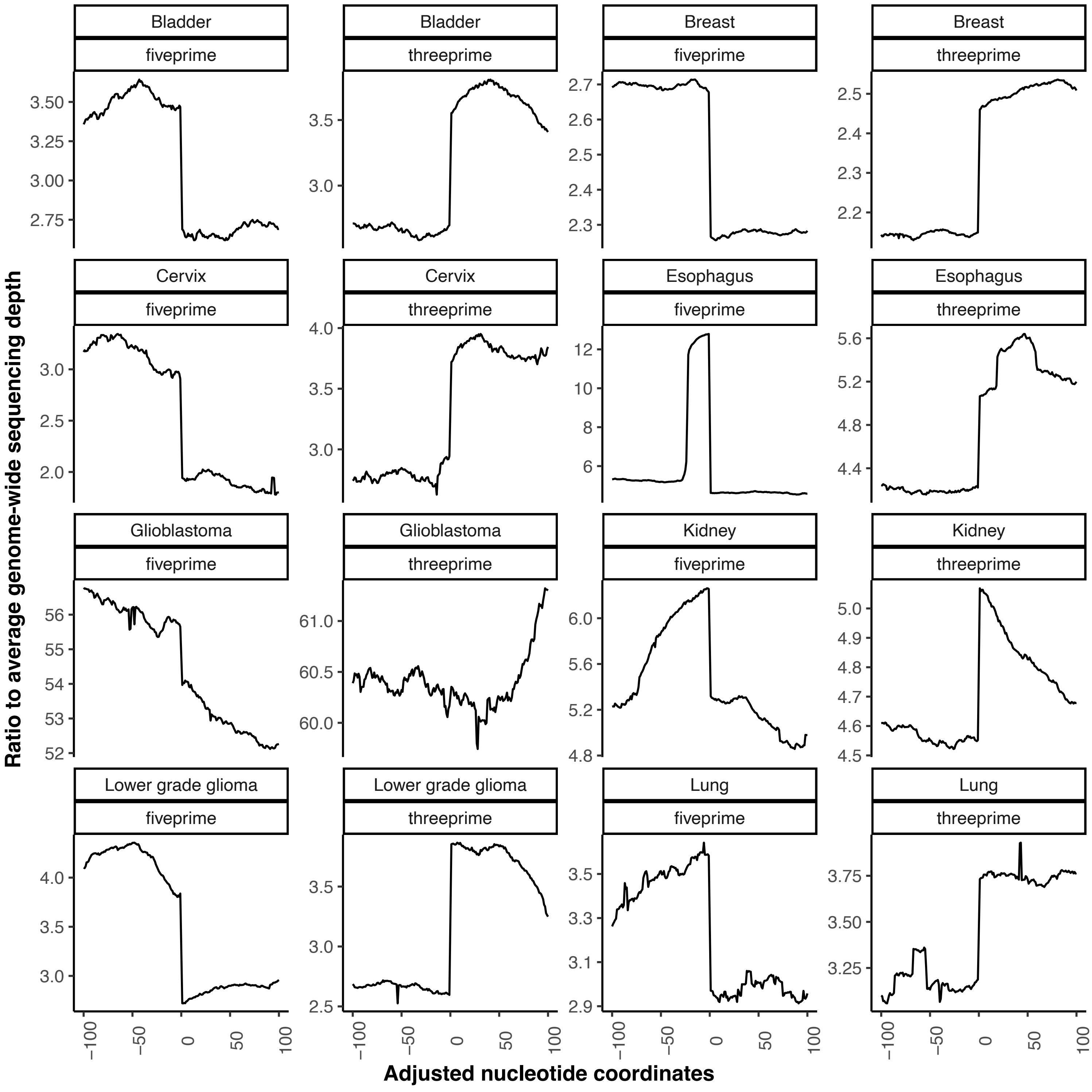

Additional file 6. Sequencing depth around genomic breakpoints in WGS data, expressed as a ratio to the genome average.
