# Supplementary material for: Improved detection of clinically relevant fusion transcripts in cancer by machine learning classification
Source: BMC Genomics. 2023 Dec 18;24:783. doi: 10.1186/s12864-023-09889-y (PMC10726539; doi:10.1186/s12864-023-09889-y)
Supplement: Supplementary file 7 — Additional file 7. Comparison of FusionCatcher with Arriba and STAR-Fusion. A Sensitivity for individual fusion prediction algorithms and ensemble predictions based on TCGA data (BRCA and LUAD combined). Upset plots for validated fusions in B the TCGA data and C the TNBC validation set. [file 12864_2023_9889_MOESM7_ESM.pdf]

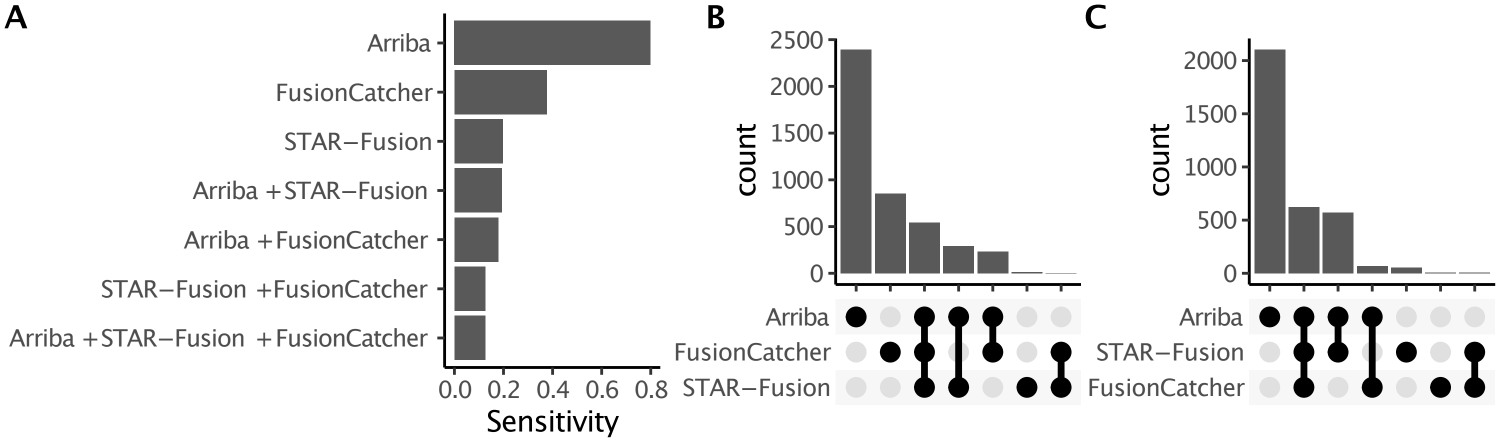

Additional file 7. Comparison of FusionCatcher with Arriba and STAR-Fusion. **A** Sensitivity for individual fusion prediction algorithms and ensemble predictions based on TCGA data (BRCA and LUAD combined). Upset plots for validated fusions in **B** the TCGA data and **C** the TNBC validation set.
